# Late-life depression, subjective cognitive decline, and their additive risk in incidence of dementia: A nationwide longitudinal study

**DOI:** 10.1371/journal.pone.0254639

**Published:** 2021-07-14

**Authors:** Sheng-Min Wang, Kyung-do Han, Nak-Young Kim, Yoo Hyun Um, Dong-Woo Kang, Hae-Ran Na, Chang-Uk Lee, Hyun Kook Lim

**Affiliations:** 1 Department of Psychiatry, Yeouido St. Mary’s Hospital, College of Medicine, The Catholic University of Korea, Seoul, Korea; 2 Department of Statistics and Actuarial Science, Soongsil University, Seoul, Republic of Korea; 3 Department of Psychiatry, Geyo Hospital, Uiwang, Korea; 4 Department of Psychiatry, St. Vincent Hospital, Suwon, Korea; 5 College of Medicine, The Catholic University of Korea, Seoul, Korea; 6 Department of Psychiatry, Seoul St. Mary’s Hospital, College of Medicine, The Catholic University of Korea, Seoul, Korea; University of New South Wales, AUSTRALIA

## Abstract

**Objective:**

Late-life depression and subjective cognitive decline (SCD) are significant risk factors for dementia. However, studies with a large sample size are needed to clarify their independent and combined risks for subsequent dementia.

**Methods:**

This nationwide population-based cohort study included all individuals aged 66 years who participated in the National Screening Program between 2009 and 2013 (N = 939,099). Subjects were followed from the day they underwent screening to the diagnosis of dementia, death, or the last follow-up day (December 31, 2017).

**Results:**

Depressive symptom presentation, recent depressive disorder, and SCD independently increased dementia incidence with adjusted hazard ratio (aHR) of 1.286 (95% CI:1.255–1.318), 1.697 (95% CI:1.621–1.776), and 1.748 (95% CI: 689–1.808) respectively. Subjects having both SCD and depression had a higher risk (aHR = 2.466, 95% CI:2.383–2.551) of dementia than having depression (aHR = 1.402, 95% CI:1.364–1.441) or SCD (aHR = 1.748, 95% CI:1.689–1.808) alone.

**Conclusions:**

Depressive symptoms, depressive disorder, and SCD are independent risk factors for dementia. Co-occurring depression and SCD have an additive effect on the risk of dementia; thus, early intervention and close follow up are necessary for patients with co-occurring SCD and depression.

## 1. Introduction

Dementia is a neurodegenerative disorder characterized by progressive cognitive impairment, behavioral disturbances, and a loss of daily function [1]. The global incidence of dementia was 50 million in 2018 and is estimated to increase to 152 million by 2050 [2]. Pharmacological treatments have been largely ineffective in modifying the disease; thus, focus has shifted to the prevention of dementia by identifying modifiable risk factors in individuals at increased risk of developing the disease [3–7].

Late-life depression is an important risk factor for dementia [8]. The temporal association between cognitive and depressive symptoms varies widely in older adults. Cognitive decline may be an initial presenting symptom in patients with depression, whereas depressive symptoms may be the earliest sign of dementia [9,10]. Nevertheless, depressive illness has been shown to play an important role in the development of dementia [11,12]. A previous meta-analysis found that a history of depression increased the risk of dementia twofold [13]. Moreover, population-based cohort studies have repeatedly shown that depression is an independent risk factor for vascular dementia (VD) and Alzheimer’s disease (AD) [14–16].

Subjective cognitive decline (SCD), which is common in older adults, may also increase the risk of dementia [17]. SCD is characterized by self-perceived worsening in cognition without objective cognitive deficits [18]. Large community-based studies have reported SCD prevalence rates as high as 50–60% in older adults, and the prevalence increases with age [19]. A number of patients with SCD show signs of preclinical AD, and SCD has been prospectively linked to underlying AD pathology [18,20]. Recent longitudinal cohort studies have shown that the risks of mild cognitive impairment (MCI) and dementia are increased fourfold and sixfold, respectively, in patients with SCD [21,22]. Moreover, SCD is associated with depression, which may further increase the risk of dementia [23–25].

Several studies have suggested that depression and SCD are prodromes of dementia; [26,27] however, the combined effect, or additive risk, of SCD and late-life depression for dementia remains unclear. Furthermore, because SCD and depression commonly co-occur in older adults, it is unclear whether they are independent risk factors for dementia [18,28].

A recent large cohort study found that depression and SCD alone were independent risk factors for dementia, and that the risk of depression increased in individuals with co-occurring depression and SCD [29]. However, the investigators used the geriatric depression scale (GDS) cut-off score to define depression, rather than definitive diagnostic criteria. Despite the fact that depressive symptoms and depressive disorder are distinct entities, the previous study did not investigate their different impacts on the risk of dementia. Similarly, the previous study defined SCD based on a single question related to memory, which does not capture the full range of memory and non-memory domains of SCD [18,25].

We investigated the effects of recent late-life depression and SCD on the risk of subsequent dementia in a large nationwide study using health insurance claims data. We tested two sequential hypotheses: depressive symptoms, depressive disorder, and SCD are independent risk factors for dementia; and the incidence of dementia will be higher in individuals with co-occurring depressive symptoms, depressive disorder, and SCD than in those with depressive disorders or SCD alone.

## 2. Methods

### 2.1. Data source

The Korean National Health Insurance (KNHI) service is a mandatory public health insurance system that offers comprehensive medical coverage to all residents of South Korea (hereafter Korea) [30]. The KNHI service manages all insurance claims in the National Health Information Database, which consists of healthcare data such as health screening data, sociodemographic variables, and mortality data for the entire Korean population. The database has been widely used in various epidemiological studies and is described in detail elsewhere [31–33].

The KNHI offers the National Health Screening Program (NHSP) every two years to all individuals aged 40 years and older [34]. The NHSP includes a questionnaire covering health-related lifestyle and medical history, basic physical measurements (e.g., body mass index and blood pressure), and clinical tests. Additionally, the National Screening Program for Transitional Ages (NSPTA) is offered to those aged 66 years, which is considered as transitional to elderly status. The NSPTA assesses cognitive function and depressive symptoms in addition to the routine NHSP data. The Korean Dementia Screening Questionnaire-P (KDSQ-P) was used to assess cognitive function [35], and Depressive Symptoms Questionnaire (DSQ) was utilized to measure depressive symptoms [36].

### 2.2. Study population

The study included individuals aged 66 years who participated in the NSPTA program between 2009 and 2013. To restrict our investigation to the association between recently diagnosed late-life depressive disorder and the incidence of dementia, patients diagnosed with a depressive disorder 12 months or more prior to the health screening were considered to have a history of depressive disorder and were excluded from the study (washout for depression). The remaining participants were classified as having a depressive disorder (DD_Yes) if they had been diagnosed with a major depressive disorder (ICD-10, F32x or F33x) within 12 months of the health screening or as having no depressive disorders (DD_No) if they had never been diagnosed with a major depressive disorder.

We used DSQ to define presence of depressive symptoms. The DSQ includes three questions derived from GDS [36] which assess depressive symptoms related to activity and feelings of worthlessness and hopelessness: “Have you dropped many of your activities and interests?”, “Do you feel pretty worthless the way you are now?”, and “Do you feel that your situation is hopeless?” Subjects who answered “no” to all three questions were classified as not having depressive symptoms (DS_No), and those who answered “yes” to one or more items were defined as having depressive symptoms (DS_Yes).

**Fig 1:**
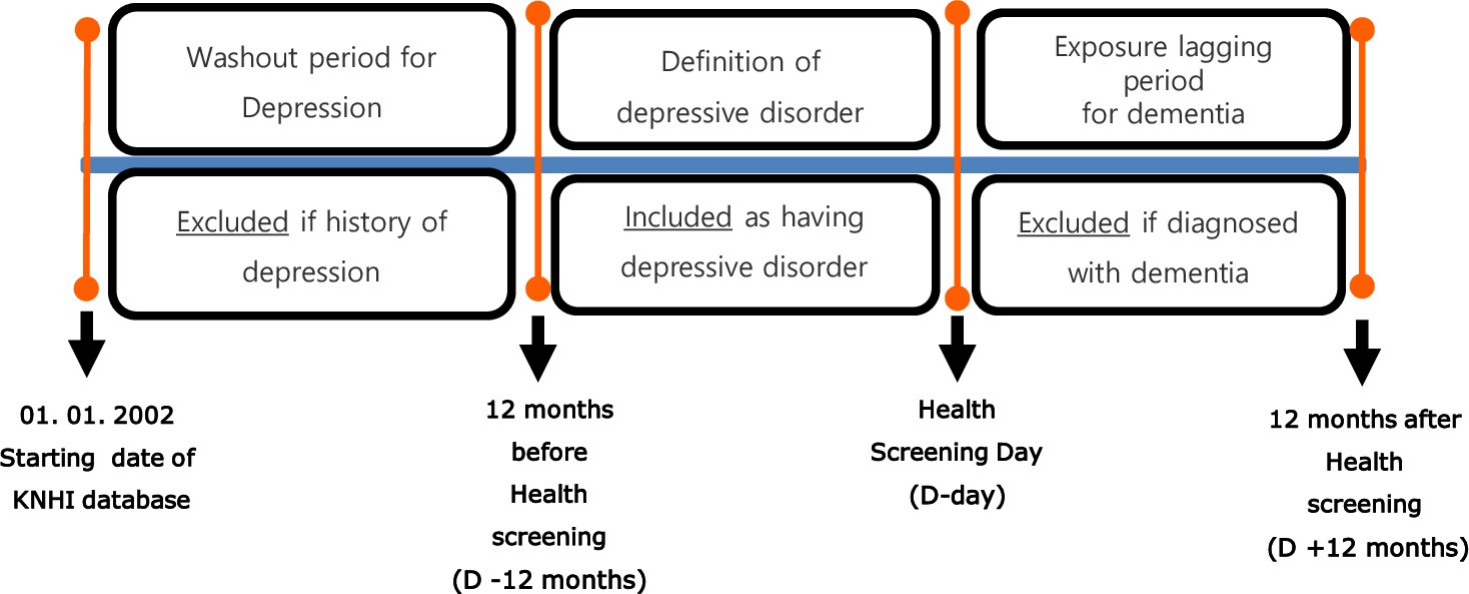
Definition of depressive disorder, washout for dementia and depressive disorder, and exposure lagging for dementia (KNHI: Korean National Health Insurance).

Subjects diagnosed with dementia (ICD-10 F00-F03, G30, or G31), MCI (ICD-10 code F067), or receiving pharmacological treatment for dementia (acetylcholinesterase inhibitors or N-methyl-D-aspartate receptor antagonists) any time before the health screening were excluded from the study (washout for dementia and MCI). Furthermore, we used an exposure lag period of 1 year to control for protopathic bias; thus, patients diagnosed with or receiving treatment for dementia within 1 year after health screening were excluded from the study (Fig 1) [37].

We assessed SCD using the KDSQ-P, a five-item self-report measure used to identify SCD in elderly individuals. The KDSQ-P is scored as 0 (No), 1 (Yes or Sometimes), and 2 (Yes or Very often). The questions include “Do you feel that your memory is worse than that of your peer/friends?, “Do you feel that your present memory is worse than it was last year?”, “Do you feel that your memory decline has had a significant impact on important activities/work?”, “Do you feel that others notice your memory loss?”, and “Do feel that you can no longer function as before due to memory loss?” [38] Subjects who answered “yes” (score 1 or 2) to four or more of the five questions were classified as having SCD (SCD_Yes), and those who answered “yes” to three or fewer of the five questions were classified as not having SCD (SCD_No).

All procedures performed in this study complied with the ethical standards of the relevant national and institutional committees on human experimentation and with the Helsinki Declaration of 1975 as revised in 2008. All procedures involving human subjects were approved by the Institutional Review Board of Yeouido St. Mary’s Hospital, Seoul, Korea (SC19ZESI0124). Consent from individual subjects was waived because the study used publicly available, deidentified data.

### 2.3. Outcome variables

The criteria for the diagnosis of dementia included: an objective measure of cognitive decline (Mini-Mental State Examination score ≤ 26) with evidence of functional impairment due to cognitive decline (Clinical Dementia Rating ≥ 1 or Global Deterioration Scale score ≥ 4), and coded for dementia in the hospital (ICD-10: F00, F01, F02, F03, G30, F051, or G311). Patients with an ICD-10 code for AD (F00 and G30) or VD (F01) were defined as incidences of AD or VD, respectively.

### 2.4. Statistical analysis

Baseline demographic and clinical characteristics were compared between groups using Student’s *t*-test for continuous variables and the chi-square test for categorical variables. Retrospective cohorts were followed from the day they underwent NSPTA health screening to the development of dementia, death, or the last follow-up day (December 31, 2017), whichever came first. The time-to-event was defined as the duration from study recruitment, the day they underwent NSPTA assessment, to the diagnosis of dementia. Cox proportional-hazard regression models were used to determine the risks of total dementia, AD, and VD related to depressive symptoms, depressive disorder, and SCD. The Cox proportional-hazard model was adjusted for potential confounding variables associated with the risk of dementia, including age, sex, smoking, alcohol drinking, physical exercise, income, body mass index, diabetes, hypertension, hyperlipidemia, and KDSQ-P. All statistical tests were performed using SAS version 9.3 (SAS Institute, Cary, NC, USA). *P*-values < 0.05 were deemed to indicate statistical significance.

## 3. Results

### 3.1. Participant characteristics

**Fig 2:**
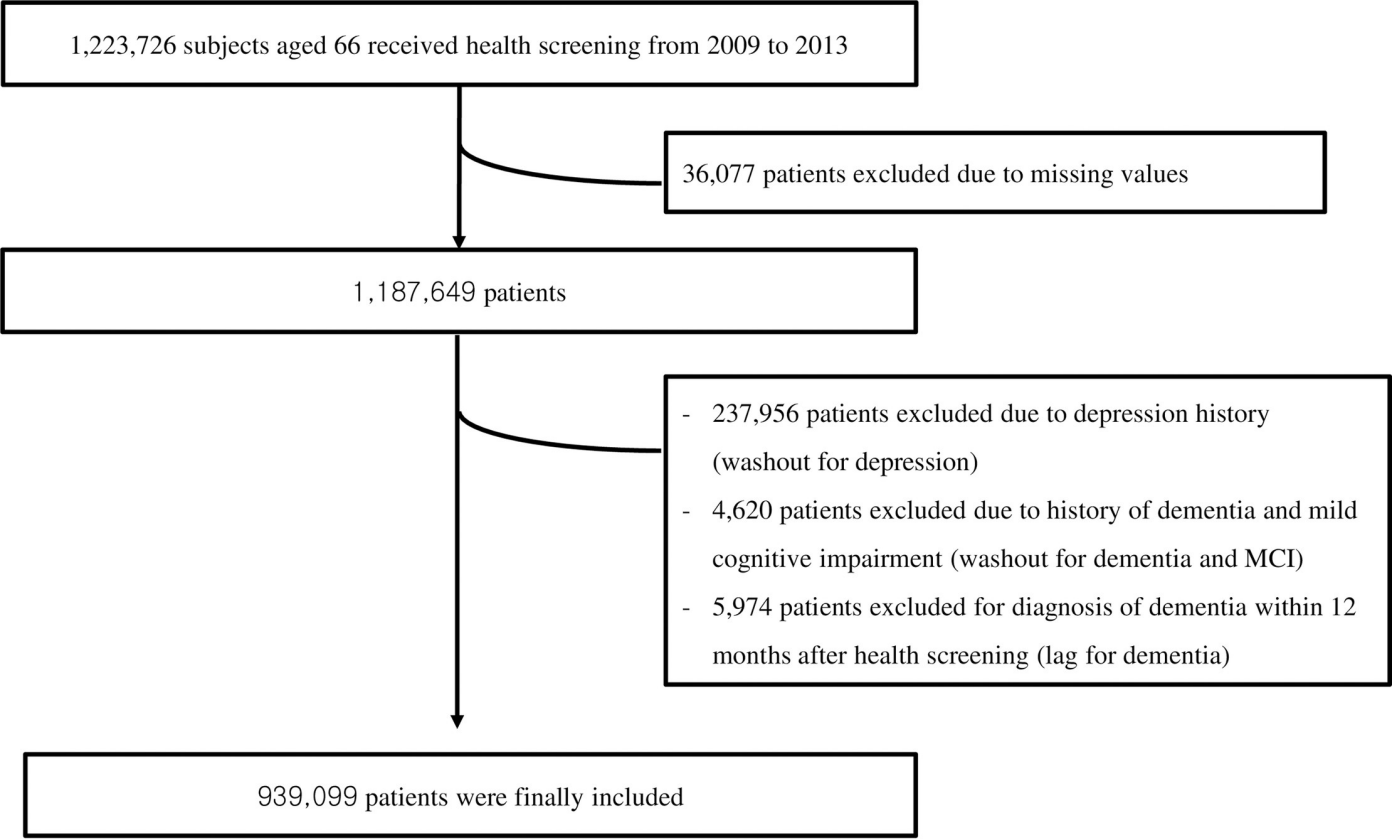
Flow chart depicting creation of study cohorts.

In total, 1,223,726 66-year-old individuals underwent health screening between 2009 and 2013. Of those, 36,077 were excluded from the study due to missing values. In addition, 237,956 participants who had a history of depressive disorder 12 months or more before the health screening (washout for depression), 4,620 participants who had a history of dementia or MCI before the health screening (washout for dementia and MCI), and 5,974 participants who were diagnosed with dementia within 12 months of the health screening (exposure lag for dementia), were excluded from the study. Of the 939,099 participants included in the final analysis (Fig 2), 79.3% (745,090) did not have a depressive disorder or depressive symptoms (non-depression group: DD_No and DS_No), whereas 20.7% (194,009) were diagnosed with a depressive disorder within 12 months of health screening or with depressive symptoms at the time of the health screening (depression group: DD_Yes or DS_Yes). The depression and non-depression groups did not differ in age as all were enrolled at the age of 66 years. The depression group had a higher percentage of females, smokers, and participants in lower income brackets. Furthermore, subjective memory complaints, the incidence of diabetes, hypertension, hyperlipidemia, vision problems, hearing impairments, and triglyceride levels were higher in the depression group than in the non-depression group. However, there were fewer alcohol drinkers in the depression group than in non-depression group (Table 1).

**Table 1:** Demographic and clinical data of patients who received mandatory public health screening at age of 66 years (total N = 939,099).

	Depression		
	No^1^	Yes^2^	p
Number of subjects	745,090 (79.3%)	194,009 (20.7%)	
SEX			< .0001
Male	383,673(51.49%)	80,477(41.48%)	
Female	361,417(48.51%)	113,532(58.52%)	
Age	66±0	66±0	
SCD (yes to 4 or more of KDSQ-P)	70,885/745,090 (9.51%)	48,277/194,009 (24.88%)	< .0001
Weight(kg)	62.06±9.58	60.94±9.57	< .0001
Height(cm)	159.7±8.36	158.3±8.27	< .0001
BMI(kg/m^2^)	24.29±2.98	24.29±3.14	0.9767
Waist Circumference	83.22±8.13	83.11±8.42	< .0001
Smoking			< .0001
Never	500,101(67.19%)	133,816(69.12%)	
Ex-smoker	141,094(18.96%)	31,846(16.45%)	
Current Smoker	103,075(13.85%)	27,943(14.43%)	
Alcohol Drinking			< .0001
No	508,840(68.62%)	140,985(73.09%)	
Mild	199,605(26.92%)	43,775(22.69%)	
Heavy	33,066(4.46%)	8,129(4.21%)	
Exercise(yes)	362,182(48.71%)	81,744(42.28%)	< .0001
Heavy	153,974(20.69%)	31,791(16.43%)	< .0001
Moderate	196,827(26.48%)	41,771(21.62%)	< .0001
Light	402,274(54.1%)	96,680(49.98%)	< .0001
Income(low)	213,398(28.64%)	56,603(29.18%)	< .0001
Diabetes(yes)	143,564(19.28%)	41,852(21.59%)	< .0001
Hypertension(yes)	395,187(53.09%)	105,774(54.57%)	< .0001
Hyperlipidemia(yes)	254,763(34.21%)	73,927(38.14%)	< .0001
TG (in Log value)	119.89(119.75–120.03)	121.7(121.42–121.97)	< .0001
Visual acuity problem(yes)	9,867(1.32%)	3,040(1.57%)	< .0001
Hearing impairment(yes)	9,333(1.25%)	2,803(1.44%)	< .0001
KDSQ-P			
Item 1			< .0001
0	587,158(78.8%)	110,433(56.92%)	
1	148,573(19.94%)	72,511(37.38%)	
2	9,359(1.26%)	11,065(5.7%)	
Item 2			< .0001
0	514,183(69.01%)	86,915(44.8%)	
1	220,339(29.57%)	94,049(48.48%)	
2	10,568(1.42%)	13,045(6.72%)	
Item 3			< .0001
0	616,189(82.7%)	125,346(64.61%)	
1	123,059(16.52%)	61,114(31.5%)	
2	5,842(0.78%)	7,549(3.89%)	
Item 4			< .0001
0	663,711(89.08%)	147,739(76.15%)	
1	77,900(10.46%)	41,946(21.62%)	
2	3,479(0.47%)	4,324(2.23%)	
Item 5			< .0001
0	625,596(83.96%)	123,034(63.42%)	
1	115,523(15.5%)	646,04(33.3%)	
2	3,971(0.53%)	6,371(3.28%)	

^1^ No depressive disorder and no depressive symptoms.

^2^ Either diagnosed with depressive disorder within 12 months prior to health screening or had one or more of the three depressive symptoms at the time of health screening BMI: Body mass Index; KDSQ-P: Prescreening Korean Dementia Screening Questionnaire; TG: Triglyceride.

### 3.2. Risk of dementia associated with depressive symptoms, depressive disorder, and SCD

With mean follow-up of 5.19 ± 1.6 (range; 0–8) years, a total of 36,268 patients were diagnosed with dementia (N = 25,277 for no depression group and N = 10,991 for depression group). Cox regression analysis revealed that the depression group (5.67%) had a higher risk of total dementia than the non-depression group (3.39%) (adjusted hazard ratio [aHR] = 1.345; 95% confidence interval [CI] = 1.314–1.377; Table 2). In terms of dementia subtypes, the risks for AD (aHR = 1.356; 95% CI = 1.32–1.393) and VD (aHR = 1.254; 95% CI: 1.176–1.337) were higher in the depression group than in the non-depression group. Patients with recent depressive disorder (DD_Yes) had a higher risk of total dementia (aHR = 1.697; 95% CI: 1.621–1.776), AD (aHR = 1.754; 95% CI: 1.665–1.847), and VD (aHR = 1.365; 95% CI: 1.192–1.563) than did those with no history of a depressive disorder (DD_No). Similar trends were observed in participants with depressive symptoms compared with those with no depressive symptoms at the time of the health screening (DS_Yes vs. DS_No).

**Table 2:** Risk of total dementia, AD, and VD according to presence of depressive symptom or depressive disorder.

	N	Dementia	Duration^a^	IR^b^	MODEL 1	MODEL 2	MODEL 3	
							Adjusted HR	P-value
**Dementia (Total)**								
Depression_No	745090	25277	3858004	6.5518	1(ref.)	1(ref.)	1 (ref.)	-
Depression_Yes	194009	10991	1017799	10.7988	1.607(1.571,1.644)	1.551(1.516,1.587)	1.345 (1.314, 1.377)	P < 000.1
Depressive symptoms								
DS_No	766722	26517	3966523	6.6852	1(ref.)	1(ref.)	1 (ref.)	-
DS_Yes	172377	9751	909280	10.7239	1.559(1.523,1.596)	1.505(1.47,1.541)	1.286 (1.255, 1.318)	P < 000.1
Depressive disorder								
DD_No	909345	34258	4726137	7.2486	1(ref.)	1(ref.)	1 (ref.)	-
DD_yes	29754	2010	149668	13.4298	1.84(1.759,1.925)	1.779(1.7,1.862)	1.697 (1.621, 1.776)	P < 000.1
**AD**								
Depression_No	745090	18699	3858004	4.84681	1(ref.)	1(ref.)	1 (ref.)	-
Depression_Yes	194009	8249	1017799	8.10474	1.617(1.575,1.66)	1.565(1.524,1.607)	1.356 (1.32, 1.393)	P < 000.1
Depressive symptoms								
DS_No	766722	19651	3966523	4.95421	1(ref.)	1(ref.)	1 (ref.)	-
DS_Yes	172377	7297	909280	8.02502	1.561(1.519,1.603)	1.51(1.469,1.552)	1.288 (1.252, 1.325)	P < 000.1
Depressive disorder								
DD_No	909345	25402	4726137	5.3748	1(ref.)	1(ref.)	1 (ref.)	-
DD_yes	29754	1546	149668	10.3296	1.897(1.801,1.997)	1.838(1.745,1.936)	1.754 (1.665, 1.847)	P < 000.1
**VD**								
Depression_No	745090	3643	3858004	0.94427	1(ref.)	1(ref.)	1 (ref.)	-
Depression_Yes	194009	1419	1017799	1.39418	1.477(1.388,1.571)	1.415(1.329,1.506)	1.254 (1.176, 1.337)	P < 000.1
Depressive symptoms								
DS_No	766722	3782	3966523	0.95348	1(ref.)	1(ref.)	1 (ref.)	-
DS_Yes	172377	1280	909280	1.40771	1.473(1.382,1.57)	1.412(1.324,1.506)	1.239 (1.159, 1.324)	P < 000.1
Depressive disorder								
DD_No	909345	4838	4726137	1.02367	1(ref.)	1(ref.)	1 (ref.)	-
DD_yes	29754	224	149668	1.49665	1.49(1.303,1.704)	1.425(1.244,1.631)	1.365 (1.192, 1.563)	P < 000.1

Model 1: Sex.

Model 2: Sex, smoking, alcohol drinking, physical exercise, income, body mass index, diabetes, hypertension, and hyperlipidemia.

Model 3: Sex, smoking, alcohol drinking, physical exercise, income, body mass index, diabetes, hypertension, hyperlipidemia, and KDSQ-P.

^a^ Person-years

^b^ Incidence Rate per 1000.

AD: Dementia due to Alzheimer’s disease; DD_No: No depressive disorder; DD_Yes: Diagnosed with depressive disorder within 12 months before health screening; DS_No: No depressive symptoms; DS_Yes: Having at least one of the three depressive symptoms; KDSQ-P: Prescreening Korean Dementia Screening Questionnaire; VD: Vascular dementia.

KDSQ-P is a scale consisting of 0 ~ 10 points, so we conducted an additional Cox regression with KDSQ-P or severity of subjective memory complaint as a continuous variable. The results showed that subjects with a higher KDSQ-P score showed a higher risk for subsequent dementia suggesting that severity of subjective memory complaint was significantly associated with risk of subsequent dementia (Table 3).

**Table 3:** Risk of total dementia according to severity of subjective memory complaints.

Total KDSQ-P score	Adjusted HR-I^a^ (95% CI)	P-value	Adjusted HR-II^b^ (95% CI)	P-value
0	1 (ref.)	-	1 (ref.)	
1	1.066 (1.032, 1.102)	P < 0.001	1.051 (1.017, 1.087)	P < 0.001
2	1.277 (1.235, 1.323)	P < 0.001	1.243 (1.2, 1.286)	P < 0.001
3	1.422 (1.365, 1.48)	P < 0.001	1.358 (1.304, 1.415)	P < 0.001
4	1.737 (1.664, 1.813)	P < 0.001	1.633 (1.564, 1.706)	P < 0.001
5	1.916 (1.849, 1.984)	P < 0.001	1.817 (1.753, 1.882)	P < 0.001
6	2.582 (2.393, 2.785)	P < 0.001	2.322 (2.151, 2.507)	P < 0.001
7	3.025 (2.762, 3.313)	P < 0.001	2.681 (2.446, 2.939)	P < 0.001
8	3.612 (3.254, 4.011)	P < 0.001	3.183 (2.865, 3.537)	P < 0.001
9	4.316 (3.8, 4.901)	P < 0.001	3.753 (3.301, 4.265)	P < 0.001
10	4.56 (4.179, 4.976)	P < 0.001	4.038 (3.698, 4.41)	P < 0.001

^a^ Adjusted for Sex, smoking, alcohol drinking, physical exercise, income, body mass index, diabetes, hypertension, and hyperlipidemia.

^b^ Adjusted for Sex, smoking, alcohol drinking, physical exercise, income, body mass index, diabetes, hypertension, hyperlipidemia, and Depressive Symptoms Questionnaire.

HR: Hazard Ratio; KDSQ-P: Prescreening Korean Dementia Screening Questionnaire.

The risk of dementia was further stratified according to the presence of depressive symptoms, recent depressive disorder, and SCD (Table 4). The risk of total dementia increased sequentially from no SCD or depression (3.14%; aHR = 1), to depression (4.73%; aHR = 1.402, CI: 1.364–1.441) or SCD alone (5.79%; aHR = 1.748, CI: 1.689–1.808), to co-occurring SCD and depression (having depressive symptoms or recently diagnosed with depressive disorder (8.47%; aHR = 2.466, CI: 2.383–2.55). Similar trends were observed for the risks of AD and VD.

**Table 4:** Combined risk of total dementia due to depressive symptom, depressive disorder history, and subjective cognitive decline.

	N	Dementia (total)	Duration^a^	IR per 1000	MODEL 1	MODEL 2
SCD_No AND (DS_No + DD_No)	674205	21173	3478885	6.0861	1(ref.)	1(ref.)
SCD_No AND (DS_Yes or DD_Yes)	145732	6901	761458	9.0629	1.456(1.417,1.497)	1.402(1.364,1.441)
SCD_Yes AND (DS_No + DD_No)	70885	4104	379119	10.8251	1.715(1.658,1.774)	1.748(1.689,1.808)
SCD_Yes AND (DS_Yes or DD_Yes)	48277	4090	256341	15.9553	2.517(2.433,2.603)	2.466(2.383,2.551)
	N	AD	Duration^a^	IR per 1000	MODEL 1	MODEL 2
SCD_No AND (DS_No + DD_No)	674205	15629	3478885	4.492	1(ref.)	1(ref.)
SCD_No AND (DS_Yes or DD_Yes)	145732	5181	761458	6.804	1.47(1.424,1.517)	1.419(1.374,1.465)
SCD_Yes AND (DS_No + DD_No)	70885	3070	379119	8.097	1.725(1.659,1.794)	1.756(1.688,1.826)
SCD_Yes AND (DS_Yes or DD_Yes)	48277	3068	256341	11.968	2.525(2.428,2.625)	2.478(2.383,2.578)
	N	VD	Duration^a^	IR per 1000	MODEL 1	MODEL 2
SCD_No AND (DS_No + DD_No)	674205	3109	3478885	0.893	1(ref.)	1(ref.)
SCD_No AND (DS_Yes or DD_Yes)	145732	914	761458	1.200	1.345(1.248,1.448)	1.283(1.191,1.383)
SCD_Yes AND (DS_No + DD_No)	70885	534	379119	1.408	1.55(1.413,1.7)	1.594(1.453,1.749)
SCD_Yes AND (DS_Yes or DD_Yes)	48277	505	256341	1.970	2.194(1.996,2.412)	2.149(1.954,2.363)

MODEL 1: Sex.

MODEL 2: Sex, smoking, alcohol drinking, physical exercise, income, body mass index, diabetes, hypertension, and hyperlipidemia.

^a^ Person-years.

AD: Dementia due to Alzheimer’s disease; DD_No: No depressive disorder; DS_Yes: Having at least one of the three depressive symptoms; IR: Incidence rate SCD_No: Having less than 3 subjective memory complaints; SCD_Yes: Having 4 or more subjective memory complaints; VD: Vascular dementia.

We also explored whether the risk of subsequent dementia differed in patients with memory dominant SCD (subjects who answered “yes” to question 1 and 2 of the KDSQ-P) and non-memory dominant SCD (subjects who answered “yes” to question 3 ~ 5 of the KDSQ-P). The results showed that the risk of subsequent total dementia, AD, and VD according to depressive symptoms and depressive disorder were generally comparable between memory dominant SCD and non-memory dominant SCD (Table 5).

**Table 5:** Risk of dementia in patients with non-memory and memory dominant SCD.

	Non-memory dominant SCD^1^				Memory dominant SCD^2^			
	Adjusted HR-I^a^ (95% CI)	P-value	Adjusted HR-II^b^ (95% CI)	P-value	Adjusted HR-I^a^ (95% CI)	P-value	Adjusted HR-II^b^ (95% CI)	P-value
**Dementia (total)**								
Depression No	1 (ref.)		1 (ref.)		1 (ref.)		1 (ref.)	
Depression Yes	1.36 (1.309, 1.414)	P < 0.0001	1.363 (1.311, 1.417)	P < 0.0001	1.45 (1.408, 1.493)	P < 0.0001	1.344 (1.304, 1.384)	P < 0.0001
DS No	1 (ref.)		1 (ref.)		1 (ref.)		1 (ref.)	
DS Yes	1.278 (1.225, 1.333)	P < 0.0001	1.28 (1.227, 1.335)	P < 0.0001	1.405 (1.364, 1.448)	P < 0.0001	1.298 (1.259, 1.337)	P < 0.0001
DD No	1 (ref.)		1 (ref.)		1 (ref.)		1 (ref.)	
DD Yes	1.783 (1.663, 1.912)	P < 0.0001	1.783 (1.663, 1.912)	P < 0.0001	1.689 (1.591, 1.793)	P < 0.0001	1.633 (1.538, 1.734)	P < 0.0001
**AD**								
Depression No	1 (ref.)		1 (ref.)		1 (ref.)		1 (ref.)	
Depression Yes	1.385 (1.324, 1.449)	P < 0.0001	1.387 (1.326, 1.451)	P < 0.0001	1.452 (1.404, 1.502)	P < 0.0001	1.347 (1.301, 1.394)	P < 0.0001
DS No	1 (ref.)		1 (ref.)		1 (ref.)		1 (ref.)	
DS Yes	1.286 (1.224, 1.35)	P < 0.0001	1.287 (1.225, 1.352)	P < 0.0001	1.404 (1.357, 1.453)	P < 0.0001	1.298 (1.253, 1.344)	P < 0.0001
DD No	1 (ref.)		1 (ref.)		1 (ref.)		1 (ref.)	
DD Yes	1.881 (1.737, 2.036)	P < 0.0001	1.881 (1.737, 2.036)	P < 0.0001	1.719 (1.605, 1.841)	P < 0.0001	1.663 (1.553, 1.781)	P < 0.0001
**VD**								
Depression No	1 (ref.)		1 (ref.)		1 (ref.)		1 (ref.)	
Depression Yes	1.214 (1.094, 1.349)	P < 0.0001	1.215 (1.094, 1.35)	P < 0.0001	1.379 (1.271, 1.495)	P < 0.0001	1.285 (1.184, 1.395)	P < 0.0001
DS No	1 (ref.)		1 (ref.)		1 (ref.)		1 (ref.)	
DS Yes	1.21 (1.081, 1.354)	P < 0.0001	1.211 (1.081, 1.355)	P < 0.0001	1.358 (1.25, 1.474)	P < 0.0001	1.262 (1.161, 1.372)	P < 0.0001
DD No	1 (ref.)		1 (ref.)		1 (ref.)		1 (ref.)	
DD Yes	1.328 (1.078, 1.635)	P < 0.0001	1.327 (1.078, 1.635)	P < 0.0001	1.432 (1.198, 1.711)	P < 0.0001	1.388 (1.161, 1.659)	P < 0.0001

^1^ Answering “yes’ to question 3 ~ 5 of the KDSQ-P.

^2^ Answering “yes” to question 1 and 2 of the KDSQ-P.

No depressive disorder and no depressive symptoms.

^a^ Adjusted for Sex, smoking, alcohol drinking, physical exercise, income, body mass index, diabetes, hypertension, and hyperlipidemia.

^b^ Adjusted for Sex, smoking, alcohol drinking, physical exercise, income, body mass index, diabetes, hypertension, hyperlipidemia, and Depressive Symptoms Questionnaire.

AD: Dementia due to Alzheimer’s disease; DD_No: No depressive disorder; DS_Yes: Having at least one of the three depressive symptoms; HR: Hazard Ratio; VD: Vascular dementia.

## 4. Discussion

We found that depressive symptoms, recent depressive disorder, and SCD independently increased the risk of dementia (total, AD, and VD) with aHRs of 1.286, 1.697, and 1.748, respectively. Furthermore, we found that co-occurring SCD and depression (recent depressive disorder or depressive symptoms) further increased the risk of subsequent dementia (aHR = 2.466).

Our findings that the percentages of females, current smokers, lower income, diabetes, hypertension, hyperlipidemia, vision problems, hearing impairment, and triglyceride levels were higher in the depression group than in the non-depression group are consistent with those of previous studies [14,15,39]. Moreover, the percentage of participants with subjective memory complaints was higher in the depression than in the non-depression group, suggesting a correlation between SCD and depression [40]. There were fewer alcohol drinkers in the depression group than in the non-depression group, suggesting a J-shaped (i.e., curvilinear) relationship between alcohol consumption and depression in elderly Koreans [41].

Our findings confirm those of previous studies that found the risk of total dementia, AD, and VD was higher in individuals with depression than in healthy control subjects [42–44]. By excluding patients with a history of depressive disorder and including only patients with recent (within 12 months before study enrollment) depressive disorder, we were able to more precisely show that recent late-life depression is associated with an increased risk of subsequent dementia. Although previous studies have shown that the risk of dementia is higher in patients with SCD [17,45], only one large cohort study by Liew et al., [29] which included 13,462 subjects, found that the risk of dementia was higher in patients with co-occurring depression and SCD than in those with depression or SCD alone. To our knowledge, ours is the largest longitudinal and first nationwide study to investigate the associations of depression and SCD with the incidence of dementia. Our longitudinal assessment of more than 930,000 subjects provides strong evidence that depression and SCD have an additive effect on the risk of dementia. Our study has certain advantages over that of Liew et al. [29]. In addition to a larger sample size, unlike Liew et al which used only 1 question focusing on memory to define SCD, we used a 5-item self-report assessing subjective memory per se and subjective feeling of functional decline due to memory deficits. Furthermore, we defined SCD as having 4 or more of the 5 SCD symptoms, so we were able to capture the full range of memory and non-memory domains of SCD [18,46].

The pathophysiological mechanisms underlying the synergistic effect of SCD and depression on the risk of dementia remain unclear. One possibility is that individuals with depression are more aware of their subjective memory problems than those without depression (reporting SCD); therefore, the participants in our depression group may have been better able to detect their cognitive decline (dementia risk) than the non-depression group. This hypothesis is supported by our finding that the prevalence of SCD was significantly higher in the participants in the depression group (37.21%) than in the non-depression group (16.22%) among participants who developed dementia. Alternatively, because the baseline prevalence of SCD was higher in the depression group (24.88%) than in the non-depression group (9.51%), depression may have been the major factor underlying the higher incidence of dementia. Additional longitudinal studies are needed to fully understand the complex causal relationships among depression, SCD, and dementia.

In terms of neurobiological pathophysiology, our results may be explained by the glucocorticoid cascade hypothesis: depression has been shown to damage the hippocampus, and the damage may be exacerbated by multiple depressive episodes [47]. However, we only included patients who were recently diagnosed with late-life depressive disorder. Thus, they did not experience long term insults to the hippocampus from depression. Hippocampal atrophy and neuroinflammation have also been observed in patients with SCD [48]. Cerebral β-amyloid deposition, which is an important pathophysiology of AD, has been known be associated with SCD [49]. Other studies suggested association between SCD with other dementias including fronto-temporal lobar degeneration, vascular dementia, and Lew body dementia [17]. Studies increasingly suggested that SCD could be one of the earliest clinical manifestations of dementia [50,51]. However, additional studies focusing on neurobiological mechanisms are needed to clarify the final common pathway linking depression and SCD to dementia.

Our study has several strengths. First, it is a nationwide study restricted to individuals aged 66 years; thus, the elimination of selection bias and minimization of an effect of recruitment setting increase the generalizability of our findings [52]. Second, we used a conservative definition of SCD, which prevented overestimation of the associations of depression and SCD with dementia. Third, we excluded patients with a history of depressive disorder (diagnosed more than 12 months before the health screening), which allowed us to specifically investigate the impact of recent depression on the risk of dementia. Finally, we investigated the incidences of AD and VD as well as that of total dementia.

Nevertheless, our study has several limitations. First, participants with cognitive complaints in the depression group could have been closer on the dementia prodrome continuum than SCD. Likewise, since it usually takes longer than 10 years for patients with SCD to develop dementia [18], some patients in our study could have been near stage of MCI than SCD. Second, dementia was diagnosed according to clinical data rather than objective cognitive test findings. Furthermore, findings reflecting amyloid burden (amyloid positron emission tomography [PET] and cerebral spinal fluid [CSF] Aß42 or Aß42/Aß40) and neuronal damage (structural magnetic resonance imaging of brain, ^18^F-FDG PET, and CSF total Tau) were not included in the analysis. We did not investigate several well-known risk factors for dementia, including the apolipoprotein E gene and education level, or the effects of antidepressants or other depression treatments on the risk of dementia. Our strict criteria for SCD (at least four of five SCD symptoms) may have resulted in underestimation of SCD prevalence and the aHR associated with dementia. Although our findings are based on data from the national health screening service, which is compulsory, there remain individuals who were not able or refused to receive health screening for various medical, geographical, and economical reasons. Almost all Koreans (> 97%) are enrolled in the mandatory health insurance, around 3% of the population is unable to pay premiums and is not covered by the mandatory health insurance [53]. Thus, our findings related to years of education, occupational attainment, and economical status may not be fully representative.

In conclusion, we found that depressive symptoms, depressive disorder, and SCD were independent risk factors for subsequent dementia. Co-occurring depression and SCD have an additive effect on the risk of dementia. Thus, early intervention and close follow up are necessary for patients with co-occurring SCD and depression.
